# Antenatal Spontaneous Renal Forniceal Rupture Presenting as an Acute Abdomen

**DOI:** 10.1155/2018/8596491

**Published:** 2018-04-10

**Authors:** Jennifer Travieso, Omar M. Young

**Affiliations:** ^1^Department of Obstetrics and Gynecology, Washington University School of Medicine, St. Louis, MO, USA; ^2^Division of Maternal-Fetal Medicine and Ultrasound, Department of Obstetrics and Gynecology, Washington University School of Medicine, St. Louis, MO, USA

## Abstract

**Background:**

Renal forniceal rupture is a lesser-known cause of acute abdomen in pregnancy. The ureteral compression by the gravid uterus places pregnant women at a higher risk. Sequelae in pregnancy could include intractable pain, acute kidney injury, and preterm birth.

**Case:**

A 22-year-old primigravida with no prior medical history presented with an acute abdomen in her second trimester. The diagnosis of renal forniceal rupture was made by a radiologist using MRI. A percutaneous nephrostomy catheter was placed, and the patient's pain was relieved. She subsequently delivered at term.

**Conclusion:**

Upon presentation of an acute abdomen in pregnancy, providers may not include renal forniceal rupture in their differential as readily as obstetric or gynecologic causes, resulting in delayed diagnosis, unnecessary invasive interventions, and potentially adverse maternal and neonatal outcomes. Increasing provider awareness could result in improved outcomes.

## 1. Introduction

Renal forniceal rupture is the extravasation of urine from one or more renal calyces caused by increased pressure in the renal collecting system [1]. The increase in pressure is secondary to obstruction, most commonly caused by nephrolithiasis. It can also be secondary to malignancy, infectious etiology, iatrogenic injury, and least commonly, pregnancy [2]. A patient may present with nondiscrete flank pain or even mimic someone with an acute surgical abdomen. Lack of provider awareness of the diagnosis has led to unnecessary open surgery [3]. In addition, untreated or undertreated forniceal rupture has been associated with preterm birth and acute kidney injury requiring hemodialysis [4–6]. The documented cases of preterm birth associated with this condition followed spontaneous preterm labor or was the result of emergent cesarean for nonreassuring fetal status. The formulation of a broad differential including conditions outside of obstetrics or gynecology is critical for providers when presented with an acute abdomen in pregnancy. We present a case of renal forniceal rupture presenting in this manner to highlight a condition where provider familiarity may be low and diligence in diagnosis is required, particularly in settings without availability of immediate radiologic diagnosis or expeditious surgical intervention.

## 2. Case

A 25-year-old primigravida with no significant past medical history at 22 weeks and 0 days gestation presented to a local hospital with sudden onset of right-sided back pain radiating to her right lower quadrant that had persisted for less than one day. She denied nausea, vomiting, fevers, vaginal bleeding, and dysuria. Her pregnancy had been otherwise uncomplicated. Her pain became uncontrollable with intravenous medication and localized solely to her right lower quadrant. She was transferred to a tertiary care center for further management, given concern for appendicitis and possible need for surgical intervention.

Her pain worsened on transport, and on arrival to the tertiary care center, she demonstrated severe right lower quadrant tenderness with rebound and voluntary guarding without costovertebral angle tenderness. She was hemodynamically stable, but intravenous hydromorphone only provided transient and mild improvement in her pain. Her cervix was found to be closed on digital exam, and no abnormalities were noted on speculum exam. Initial laboratory evaluation demonstrated a normal comprehensive metabolic panel and coagulation studies. Hemoglobin was 10.7 gm/dL and white blood cell count was not elevated (9.9 × 10^3^/*μ*L). Urinalysis was negative.

**Figure 1:**
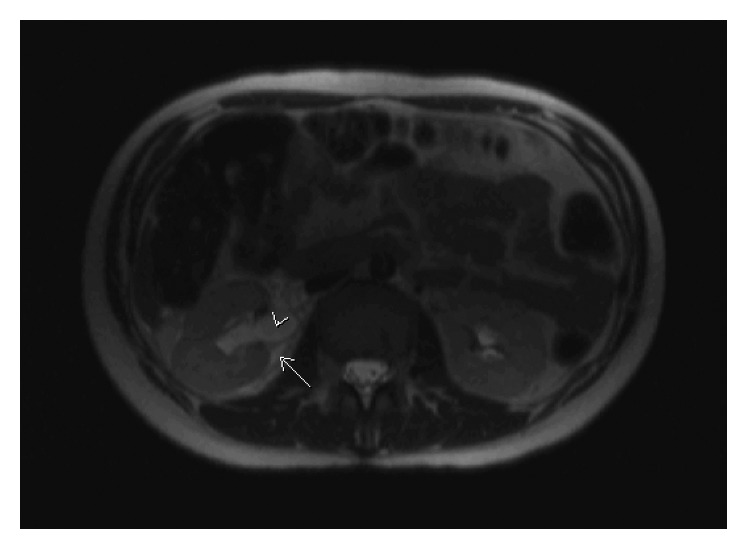
Coronal slice of MRI obtained on admission showing right hydroureter (arrowhead) and fluid extravasation surrounding the right kidney (arrow).

She underwent an abdominal MRI showing a normal appendix. However, in a verbal read from the on-call radiologist, concern was communicated for right forniceal rupture given the constellation of radiologic findings of hydroureter combined with perinephric and retroperitoneal fluid, highlighted in Figure 1. Her left kidney and collecting system were normal in appearance. Renal ultrasound was therefore performed, and it revealed right ureteral tapering between the gravid uterus and right iliac artery with no right ureteral jet visualized. Given these findings, the patient was subsequently managed by a multidisciplinary team consisting of maternal-fetal medicine, urology, and interventional radiology. Three strategies were discussed and included conservative management with close follow-up, placement of a ureteral stent, and placement of a percutaneous nephrostomy (PCN) tube. Patient preference was for PCN placement. Of note, urine culture collected prior to PCN placement was negative.

Following its placement by interventional radiology, her pain was relieved, and she was discharged with follow-up with maternal-fetal medicine and interventional radiology. Her pregnancy was subsequently complicated by readmission for recurrent pain and pyelonephritis with culture isolation of *Enterobacter cloacae* resistant to both nitrofurantoin and trimethoprim/sulfamethoxazole (TMP-SMX). She required placement of a midline IV for daily infusion of ertapenem at her local hospital. Her pregnancy was also complicated by fetal growth restriction, diagnosed at 35 weeks. Uncomplicated vaginal delivery occurred at 37 weeks and 1 day. Her PCN was removed postpartum at which point antibiotics were also discontinued.

## 3. Discussion

The diagnosis of renal forniceal rupture in pregnancy is not straightforward. While flank pain or costovertebral angle tenderness may prompt this consideration in the nonpregnant patient, an acute abdomen in a pregnant patient may not. The physiology of pregnancy often obscures diagnoses that are otherwise readily made. A gravid uterus displacing the appendix may result in an atypical presentation of appendicitis. Similarly, the physiologic dilation of the ureters described above can both predispose to this condition, and knowledge of this physiology can complicate radiologic interpretation. Provider awareness and diligence in creating an exhaustive differential, obtaining appropriate imaging in the stable patient as outlined below, and contacting appropriate teams for treatment can prevent unnecessary procedures and adverse outcomes in the pregnant patient with forniceal rupture.

Spontaneous renal forniceal rupture is caused by various etiologies resulting in an increase in pressure. This diagnosis is not exclusive to pregnancy, and in a retrospective review of forniceal rupture, ureteric stones were the cause in 74% of cases. Other causes included malignant (8%) or benign (2%) extrinsic compression, anatomic obstruction (4%), and iatrogenic causes (4%) [2]. While this review included one pregnant patient, another review was performed solely on pregnant patients with forniceal rupture. This review of 15 cases showed structural causes such as vesicoureteral reflux or infectious causes such as pyelonephritis and abscess as the explanation in 5 cases (31%), benign tumors as the cause in 4 cases (25%), and most significantly, no cause other than pregnancy found in 6 cases (38%) [7]. Compression of the ureters by the gravid uterus is the cause for the increase in pressure responsible for rupture. At 20 weeks of gestation, 76% of pregnant patients will demonstrate right-sided hydroureter and 36% left-sided. Anatomical differences in the courses of the ovarian veins are thought to be responsible as the right-sided vessel courses across the ureter to enter the vena cava. In contrast, the left-sided vessel runs in parallel with the ureter and enters the renal vein [8]. Correspondingly, rupture in pregnant patients occurs on the right in 88% of cases [8], whereas rupture in nonpregnant patients is right-sided only 49% of the time [2].

Presentation of an acute abdomen in pregnancy requires prompt and directed evaluation while also ensuring patient stability and fetal reassurance. The differential should be organized into the following etiological categories: obstetric/gynecologic (placental abruption, preterm labor, ectopic or heterotopic pregnancy, ovarian cysts or tumors, pelvic inflammatory disease, and degeneration of fibroid tumors), renal/urologic (nephrolithiasis, pyelonephritis, spontaneous forniceal rupture, and renal malignancy), and gastrointestinal/hepatobiliary (appendicitis, pancreatitis, gallbladder disease, inflammatory bowel disease, and bowel obstruction) [4, 7]. While spontaneous renal forniceal rupture does not invariably present as an acute abdomen, improving provider awareness of this entity can prevent delaying accurate diagnosis. Misdiagnosis has led to previously documented unnecessary invasive interventions and adverse obstetric outcomes. In a case of misdiagnosis, a primigravida patient suspected to have appendicitis underwent an urgent exploratory laparotomy, revealing a normal appendix. Further intraoperative studies were then undertaken and revealed her symptoms to have been caused by forniceal rupture [3]. In a case of delayed diagnosis, a primigravida at 33 weeks presented with threatened preterm labor and flank tenderness with sterile urine. After an unremarkable ultrasound, she was conservatively managed for renal colic, until repeated episodes of pain prompted an MRI showing calyceal rupture and leak. Stent placement was planned by urology, but rapidly progressing preterm labor resulted in delivery 3 hours later [4]. Not enough cases of rupture exist in the literature to draw a conclusion about the correlation between forniceal rupture and preterm birth, but the pathophysiology is plausible with inflammation, tissue injury, and peritoneal irritation from urine resulting in preterm labor. More research is required to determine if forniceal rupture and preterm birth are clinically correlated and if there is an underlying physiologic mechanism.

Management of forniceal rupture is straightforward and requires placement of either a ureteral stent or percutaneous nephrostomy catheter, whereas detecting the diagnosis is arguably more difficult. This is likely secondary to an understandable lack of provider awareness, given that less than 35 cases exist in the literature as recently as 2016 [3]. Additionally, radiologic diagnosis is usually required. Ideal examinations include CT with IV contrast using renal protocol or IV urogram, which are not ideal in pregnancy [1]. MRI or ultrasound has been successfully used in the cases presented [1, 4]. The diagnosis can be made by any one of the following criteria being met: irregularity of a single renal calyx, loss of the ability to discern renal sinus fat, asymmetrically distributed perinephric stranding, and a discreet perinephric fluid collection [2]. Not all centers are equipped to provide this imaging or interpretation, and in these cases, the responsibility for higher suspicion and appropriate triage falls to the provider available.

## Additional Points

Prompt diagnosis of antenatal renal forniceal rupture can prevent unnecessary interventions and adverse outcomes; however, such diagnosis requires increased provider awareness.
